# Formulation Development and Characterization of Meclizine Hydrochloride Sublimated Fast Dissolving Tablets

**DOI:** 10.1155/2014/281376

**Published:** 2014-08-25

**Authors:** Sateesh Kumar Vemula, Mohan Vangala

**Affiliations:** ^1^College of Medical and Health Sciences, Wollega University, P.O. Box No 395, Nekemte, Ethiopia; ^2^Department of Pharmaceutics, Chaitanya College of Pharmacy Education and Research, Kishanpura, Hanamkonda, Warangal, Andhra Pradesh 506001, India

## Abstract

The intention of present research is to formulate and develop the meclizine hydrochloride fast dissolving tablets using sublimation method to enhance the dissolution rate. In this study an attempt was made to fasten the drug release from the oral tablets by incorporating the superdisintegrants and camphor as sublimating agent. The prepared fast dissolving tablets were subjected to precompression properties and characterized for hardness, weight variation, friability, wetting time, water absorption ratio, and disintegration time. From *in vitro* release studies, the formulation F9 exhibited fast release profile of about 98.61% in 30 min, and disintegration time 47 sec when compared with other formulations. The percent drug release in 30 min (*Q*_30_) and initial dissolution rate for formulation F9 was 98.61 ± 0.25%, 3.29%/min. These were very much higher compared to marketed tablets (65.43 ± 0.57%, 2.18%/min). The dissolution efficiency was found to be 63.37 and it is increased by 1.4-fold with F9 FDT tablets compared to marketed tablets. Differential scanning calorimetry and Fourier transform infrared spectroscopy studies revealed that there was no possibility of interactions. Thus the development of meclizine hydrochloride fast dissolving tablets by sublimation method is a suitable approach to improve the dissolution rate.

## 1. Introduction

Meclizine hydrochloride (MCZ) is a first-generation antihistamine of the piperazine class drug, used in the treatment of motion sickness (H_1_ receptor antagonist). MCZ is a white to light yellowish-white crystalline powder and practically insoluble in water [1, 2]. Poor water soluble drugs are allied to slower rate of absorption from oral route, so, there is a necessity to enhance the dissolution of these drugs to ensure maximum therapeutic utility of these drugs [3–5]. Formulation of oral solid dosage forms is convenient for many drugs but they are challenging to formulate if the active substances have poor dissolution rate or low bioavailability. To overcome such problems various techniques have been introduced to enhance the dissolution rate and solubility of the drug [6–8].

One of the dissolution enhancement methods is the sublimation technique, which is most widely used, and industry feasible method to formulate fast dissolving tablets [9]. Sublimation has been used to produce fast dissolving tablets with high porosity. A porous matrix is formed by compressing the volatile ingredients along with other excipients into tablets, which are finally subjected to a process of sublimation [10]. Some of the recent research examples on sublimation method are ondansetron [11], lovastatin [12], and clonazepam [13]. The objective of the present study is to enhance the dissolution rate of MCZ by sublimation method with the aid of superdisintegrants. In the present study, camphor sublimated fast dissolving tablets were prepared and studied the effect of superdisintegrants on the dissolution rate of MCZ. Some of the recent research examples on MCZ are meclizine hydrochloride mouth dissolving tablets [14], cyclodextrin-meclizine HCl inclusion complexes [15], meclizine hydrochloride solid dispersions using polyethylene glycol 8000 [16], meclizine HCl orally disintegrating tablets [17], meclizine-maltodextrin orodissolving tablets [18].

## 2. Materials and Methods

### 2.1. Materials

Meclizine hydrochloride is obtained as a gift sample from Symed labs Ltd, India. Sodium starch glycolate, Crospovidone, and Croscarmellose were gift samples from Matrix laboratories, Hyderabad, India. Camphor is obtained from Qualikems Pvt. Ltd. All other chemicals used were of analytical grade.

### 2.2. Powder Characterization

Powder mixtures of different formulations were evaluated for angle of repose, bulk density, tapped density, and compressibility index. The fixed funnel method was employed to measure the angle of repose (*θ*) and it was calculated using the following formula:

(1) $$\begin{array}{c} \text{Tan}\;\theta =\frac{h}{r}, \end{array}$$

in which *θ* is the angle of repose, *h* is the height of the cone, and *r* is radius of the cone base. To measure the angle of repose, a funnel was fixed to a stand so that the lower tip of the funnel is 2.5 cm above the surface. A graph paper was placed on a flat surface. The powder blend was allowed to fall freely on the graph paper through the funnel, till the tip of the heap formed just touches the funnel. The radius of the heap was noted and from this angle of repose was determined. Angle of repose less than 30° suggests free flowing properties of the material.

The bulk density of a powder is determined by measuring the volume of a known mass of powder sample that may have been passed through a screen, into a 50 mL graduated cylinder. Tapped densities of powder samples were determined by a tap density apparatus (Intelli, Kshitij Innovations, India). The apparatus was set for 500 tappings for 5 min at a stroke height of 20 mm [19]. The compressibility index (Carr's Index) is a measure of the propensity of a powder to be compressed. It is determined from the bulk and tapped densities and is calculated using the following formulas:

(2) Carr's Index=((ρtap−ρb)ρtap)×100,

in which *ρ*_*b*_ is bulk density and *ρ*_tap_ is tapped density.

### 2.3. Preparation of Sublimated Fast Dissolving Tablets (FDTs)

Accurately weighed quantity of drug, camphor, and superdisintegrants were passed through number 60 mesh and carefully added to avicel ph 102 and mixed in a poly bag for 15 min. Then the powder mixture was lubricated with talc and magnesium stearate by blending for another 5 min. The resultant mixture was directly compressed into tablets with 6 mm round flat punches using 8-station rotary tabletting machine (Riddhi Pvt. Ltd, India). Then these tablets were subjected to sublimation, by placing in a hot air oven at 60°C for 2 h to generate a porous matrix, due to removal of volatilizable component (15, 16). The final weight of the tablet was adjusted to 100 mg and the compositions of the tablets were given in Table 1.

### 2.4. Evaluation of Fast Dissolving Tablets

The prepared tablets were studied for their physical properties like weight variation, hardness, and friability. For estimating weight variation, 20 tablets of each formulation were weighed using an electronic weighing balance (AW 120, Shimadzu Corporation, Japan). The strength of tablet is expressed by measuring hardness and friability. The hardness of six tablets was measured using Monsanto tablet hardness tester. Friability was determined on ten tablets in a Roche friabilator (Electrolab, Mumbai, India) for 4 min at 25 rpm.

### 2.5. Determination of Drug Content

For estimation of drug content, ten tablets were crushed, and the aliquot of powder equivalent to 50 mg of drug was dissolved in suitable quantity of methanol/0.1 N HCl solution. Solution was filtered and diluted and drug content was determined by UV-Visible spectrophotometer (Systronics 2202, Ahmedabad, India) at 232 nm. The drug concentration was calculated from the calibration curve.

### 2.6. *In Vitro* Disintegration Time

*In vitro* disintegration time of FDT's was determined by following the procedure described by Gohel et al. Briefly, 10 mL of water at room temperature was taken in a petri dish of 10 cm in diameter. The tablet was then carefully placed in the centre of petri dish and the time required for the tablet to completely disintegrate into fine particles was noted. Measurements were carried out in triplicates [20].

### 2.7. Wetting Time

Wetting time was determined as described in the literature elsewhere. Briefly, two circular tissue papers were placed in a petri dish of 10 cm diameter. Ten milliliter of water containing 0.5 (% w/v) of phenol red was added to the petridish. The dye solution was used to identify the complete wetting of the tablet surface. A tablet was carefully placed on the surface of the paper in the petri dish at room temperature. The time required for water to reach the upper surface of tablet and to completely wet them was noted as wetting time. Wetting time was recorded using stopwatch and the measurements were carried out in triplicates.

### 2.8. Water Absorption Ratio (*R*)

The weight of the tablet prior to placement in the petri dish was noted (*W*_*b*_) using digital balance (Shimadzu, Japan). The wetted tablet was removed and reweighed (*W*_*a*_). Water absorption ratio (*R*), was then calculated according to the following:

(3) R=Wa−WbWb×100.

### 2.9. *In Vitro* Dissolution Study

The release of MCZ from FDT was carried out using USP XXIV Type II (paddle method) dissolution apparatus (Lab India) at a rotation speed of 100 rpm and a temperature of 37 ± 0.5°C. The drug release studies were carried out in 0.1 N HCl buffer. An aliquot of 5 mL was collected at predetermined time intervals and replaced with fresh dissolution medium. The samples were filtered by passing through 0.45 *μ*m membrane filters (Millipore, USA) and analyzed spectrophotometrically at 232 nm.

### 2.10. Calculation of Dissolution Parameters

Cumulative percent drug release was plotted as a function of time and percent drug release in 30 min (*Q*_30_) was calculated. Initial dissolution rate (IDR) was calculated as percentage dissolved of drug over the first 30 min per minute. Dissolution efficiency (DE) was calculated from the area under the dissolution curve at time *t* (measured using the trapezoidal rule) and expressed as a percentage of the area of the rectangle described by 100% dissolution in the 30 min. Relative dissolution rate (RDR) is the ratio between amount of drug dissolved from best formulation and that dissolved from the marketed tablets at 30 min [21].

### 2.11. Drug-Polymer Interaction Studies

To study the possible interaction between MCZ and excipients, DSC study was carried out on pure MCZ, camphor, and best formulation. Differential thermal analysis thermograms were obtained using DSC (Perkin-Elmer, Shelton, U.S). The analyses were performed under nitrogen (nitrogen flow rate 50 mL/min) in order to eliminate oxidative and pyrolytic effects at a standard heating rate of 15°C/minute over a temperature range of 50°C–350°C. The FTIR spectra of MCZ, camphor, and best formulation recorded between 400 to 4000 cm^−1^ on FTIR to detect the drug-excipients interactions. The FTIR spectra for the test samples were obtained using KBr disk method using an FTIR spectrometer (Perkin Elmer FT-IR, Perkin Elmer Inst. USA). The resultant spectra were compared for any possible changes in the peaks of the spectra.

### 2.12. Stability Studies

To assess the drug and formulation stability, stability studies were done according to ICH guidelines. Best formulation was sealed in aluminum packaging coated inside with polyethylene, and three replicates were kept in the humidity chamber maintained at 40 ± 2°C and 75 ± 5% RH for six months [22]. Samples were collected after six months of storage and analyzed for the drug content and* in vitro* dissolution rate and they were subjected to statistical analysis using paired *t*-test to test the significance of difference at 0.05 level of significance (LS). Then the similarity index was calculated between dissolution rates of optimized tablets before and after storage to prove the stability of the dosage form.

## 3. Results and Discussion

### 3.1. Powder Characterization

The powder mixtures of different formulations were evaluated for angle of repose, bulk density, tapped density, and compressibility index and their values were shown in Table 2. Bulk density and tapped density values ranged from 0.362 to 0.427 and 0.456 to 0.545, respectively. The results of angle of repose and compressibility index (%) ranged from 29.56 to 48.42 and 11.54 to 31.25, respectively. The results of angle of repose (<30) and compressibility index (<22) indicate fair to passable flow properties of the powder mixture [23]. Appreciable flow properties facilitate the flow of powder mixture during the tabletting process.

### 3.2. Evaluation of Fast Dissolving Tablets

The physical evaluation parameters of MCZ fast dissolving tablets were shown in Table 3. In weight variation test, the pharmacopoeial limit for the tablets was found to be in the range of not more than 7.5% of the average weight. The hardness of the tablets was found to be in the range of 3.28 ± 0.08 to 3.58 ± 0.11 kg/cm^2^. Another measure of tablets strength is friability. Conventional compressed tablets that lost less than 1% of their weight are generally considered acceptable. The percentage friability for all formulations was below 1%, that is, 0.40 ± 0.01 to 0.67 ± 0.02, indicating that the friability is within the prescribed limits. The tablets were found to contain 95.8 ± 1.74 to 99.9 ± 0.70% of the labeled amount indicating uniformity of drug content. The disintegration time of all formulations was found in the range of 47 ± 2.5 to 61 ± 3.0 sec. The wetting time of formulated tablets was found in the range of 122 ± 2.5 to 146 ± 2.6 sec and water absorption ratio was 135 ± 1.87 to 203 ± 1.32. From the results it has been found that FDTs containing crospovidone as super disintegrating agent showed better results than the others. The formulation F9 containing 6% w/w crospovidone showed the fastest disintegration (47 sec) and less wetting time (122 s) as compared to other formulations. In a study, that is, formulation of piroxicam fast disintegrating tablets [24] and formulation of ondansetron fast dissolving tablet by camphor sublimation [11] similar type of results were showed with crospovidone when compared to other superdisintegrants.

### 3.3. *In Vitro* Dissolution Study

**Figure 1:**
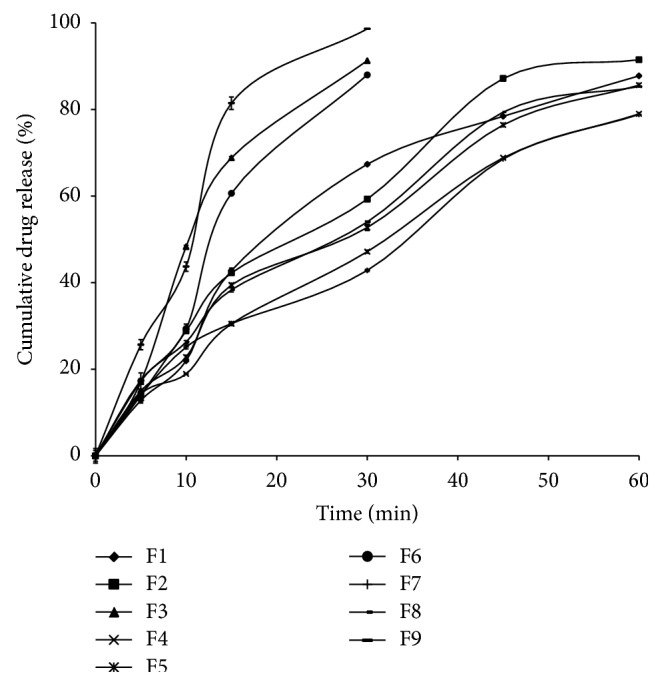
Release profile of MCZ from sublimated fast dissolving tablets (*n* = 3).

**Figure 2:**
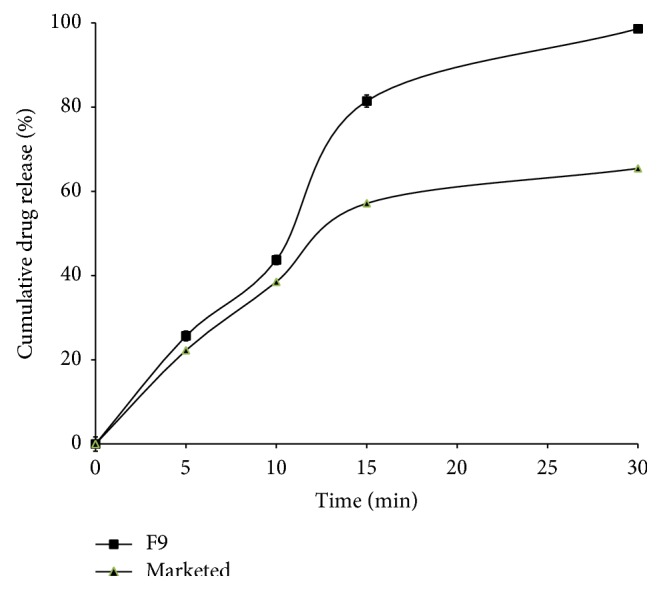
Comparison of MCZ release from F9 sublimated fast dissolving tablets and marketed tablets (*n* = 3).

From the preliminary studies to optimize the suitable sublimating agent and concentration used in the fast dissolving tablets, different formulations were prepared and evaluated for drug release using different sublimating agents in various proportions. From the dissolution studies, the formulation containing 10 mg of camphor showed fast dissolution rate with good flow properties and better tablet integrity (data is not presented). From the* in vitro* dissolution studies, tablets made from crospovidone showed faster dissolution rate than other superdisintegrants. Among all the formulations, F9 tablets showed complete drug release within 30 min and rapid dissolution when compared to other formulations, that is, 98.61 ± 0.25% in 30 min, whereas in the similar conditions the marketed tablets of same dose showed 96.09 ± 0.59% drug release in 60 min. The possible reasons and mechanisms for increased dissolution rates are formation of porous structure on the surface of tablet due to sublimation and the presence of superdisintegrants enhance the water permeation (wicking action) in to the tablet leads to fasten the wetting action, disintegration time and finally causes the fast dissolution rate [25]. The porous structure is formed due to the evaporation of sublimating agents. This porous structure enhanced the dissolution fluid in to the tablets and increased the internal pressure that leads to the fast disintegration as well as dissolution rate.  Figure 1 demonstrated the MCZ release patterns from F1 to F9 formulations and Figure 2 represents the comparison between F9 and marketed tablets. The similarity factor between F9 and marketed tablets was calculated and it was found to be 34.07 that demonstrate the significant improvement in the dissolution rate in case of F9 sublimated fast dissolving tablets.

### 3.4. Calculation of Dissolution Parameters

The percent drug release in 30 min (*Q*_30_) and initial dissolution rate (IDR) for formulation F9 was 98.61 ± 0.25%, 3.29%/min. These were very much higher compared to marketed tablets (65.43 ± 0.57%, 2.18%/min). The improvement in the dissolution characteristics of a drug described in terms of dissolution efficiency (DE) and relative dissolution rate (RDR). The RDR was found to be 1.51. The DE was found to be 63.37 and it is increased by 1.4-fold with F9 FDT tablets compared to marketed tablets, that is, 45.53 (Table 4). Similar type of improvement in IDR, DE, and RDR was reported in the study of Vemula and Veerareddy, that is, flurbiprofen fast disintegrating tablets [21].

### 3.5. Drug Polymer Interaction Studies

**Figure 3:**
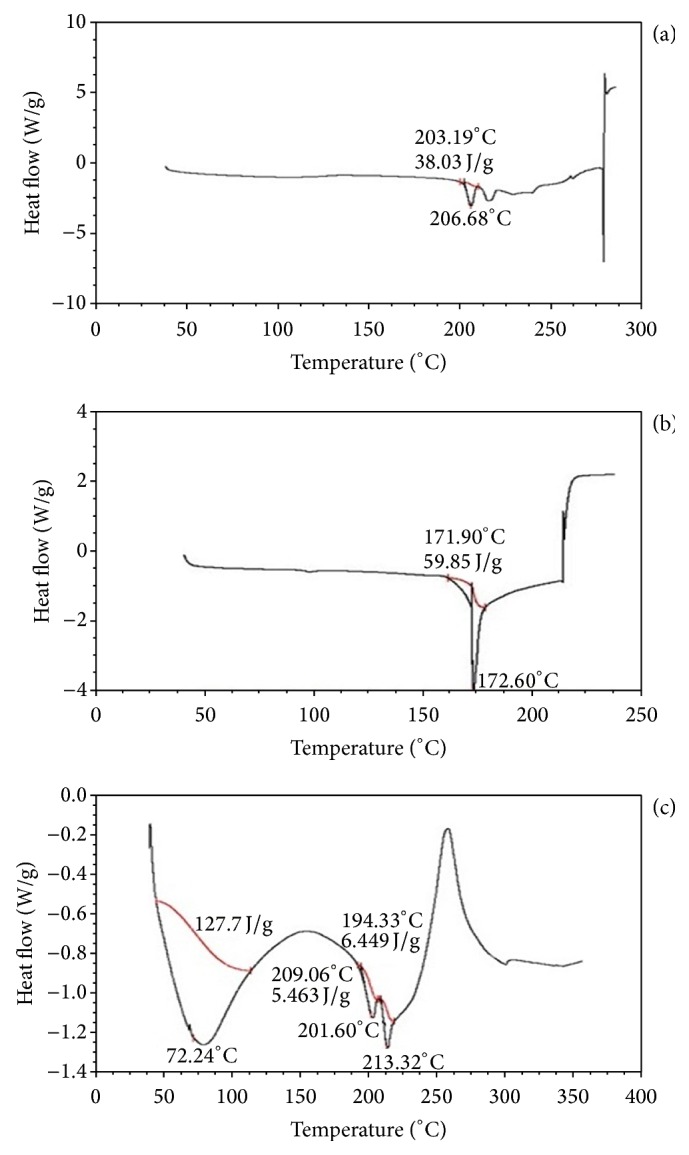
DSC thermograms of MCZ, camphor, and F9 formulation.

**Figure 4:**
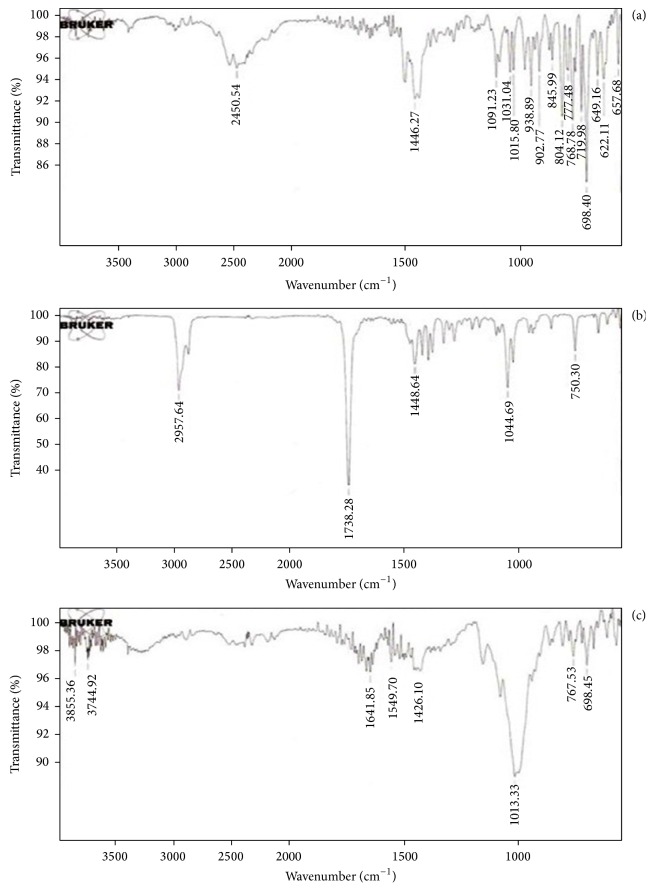
Fourier transform infrared spectra of MCZ, camphor, and F9 formulation.

DSC thermograms obtained for pure drug, camphor, and F9 formulations were shown in Figure 3. The DSC thermogram of MCZ showed endothermic peak at 206.66°C and camphor showed at 171.90°C, whereas thermogram of the F9 formulation did not show any significant shift in the endothermic peak of drug. Thermogram of the F19 formulation did not show any significant shift in the endothermic peak when compared to pure drug, indicating that there was no change in MCZ in the sublimated tablet. The FTIR spectrum of pure drug, camphor, and F9 formulations were compared in Figure 4. The interpretation of FTIR spectra was explained in Table 5. From the FTIR spectral analysis all the principal peaks observed in pure drug were present in the FTIR spectra of the F9 sublimated fast dissolving tablets and some additional peaks were observed, which could be due to the presence of camphor and other excipients. These results suggest that there is no interaction between the drug and excipients used in the present formulation study.

### 3.6. Stability Studies

To manifest the prospective utility of the formulation, stability studies were carried out at 40 ± 2°C and 75 ± 5% RH for six months. After storage of six months, the formulation F9 was subjected to a drug assay and* in vitro* dissolution studies (Table 6) and from the statistical analysis there was no significant difference between before and after storage (*P* < 0.05). The similarity index value between dissolution profiles of optimized formulation before and after storage was found to be 79.16, which is more than 50 indicates similarity between the dissolution profile before and after storage [26–28].

## 4. Conclusion

Fast dissolving tablets show better patient acceptability and compliance with improved efficacy when compared with conventional dosage forms. An attempt was made to develop the fast dissolving tablets of meclizine hydrochloride by sublimation method to achieve fast dissolving effect and to enhance the bioavailability. Meclizine hydrochloride fast dissolving tablets were successfully formulated by employing direct compression method and evaluated for different parameters, which were found in the acceptable range. From the dissolution studies of all formulations, F9 formulation showed rapid disintegration time as well as fast dissolution rate. The percent drug release in 30 min (*Q*_30_) and initial dissolution rate (IDR) for formulation F9 was 98.61 ± 0.25%, 3.29%/min. These were very much higher compared to marketed tablets (65.43 ± 0.57%, 2.18%/min). The DE was found to be 63.37 and it is increased by 1.4-fold with F9 FDT tablets compared to marketed tablets. In conclusion, development of fast dissolving tablets using sublimation method is able to enhance the dissolution rate of meclizine hydrochloride.

## Figures and Tables

**Table 1:** Formulations of meclizine hydrochloride FDTs by sublimation method.

Ingredients (mg)	F1	F2	F3	F4	F5	F6	F7	F8	F9
Meclizine HCl	25	25	25	25	25	25	25	25	25
Sodium starch glycolate	2	4	6	—	—	—	—	—	—
Croscarmelose sodium	—	—	—	2	4	6	—	—	—
Crospovidone	—	—	—	—	—	—	2	4	6
Camphor	10	10	10	10	10	10	10	10	10
Microcrystalline cellulose	59	57	55	59	57	55	59	57	55
Aspartame	1	1	1	1	1	1	1	1	1
Magnesium stearate	1	1	1	1	1	1	1	1	1
Talc	2	2	2	2	2	2	2	2	2

Total tablet weight	100	100	100	100	100	100	100	100	100

**Table 2:** Powder characterization of formulation blend.

Formulation	Bulk density (g/cc)	Tapped density (g/cc)	Angle of repose (°)	Carr's index (%)
F1	0.362	0.485	40.61	25.36
F2	0.381	0.538	48.42	28.30
F3	0.385	0.545	41.52	31.25
F4	0.410	0.475	31.20	18.47
F5	0.427	0.462	33.39	22.36
F6	0.419	0.493	37.41	24.74
F7	0.394	0.456	29.56	11.54
F8	0.424	0.477	31.25	12.24
F9	0.422	0.471	34.22	12.35

**Table 3:** Evaluation of fast dissolving tablets prepared by sublimation method.

Formulation	Weight variation (mg)∗	Hardness (kg/cm^2^)∗∗	Friability (%)∗∗∗	Disintegration time (sec)∗∗∗	Water absorption ratio∗∗∗	Wetting time (sec)∗∗∗
F1	99 ± 0.99	3.47 ± 0.21	0.40 ± 0.01	61 ± 3.0	147 ± 1.22	139 ± 3.5
F2	100 ± 1.10	3.32 ± 0.17	0.46 ± 0.02	58 ± 2.0	166 ± 1.45	133 ± 3.2
F3	101 ± 0.84	3.45 ± 0.08	0.40 ± 0.04	55 ± 1.5	135 ± 1.87	146 ± 2.6
F4	98 ± 1.37	3.50 ± 0.07	0.43 ± 0.02	56 ± 3.0	156 ± 1.32	140 ± 2.0
F5	101 ± 1.52	3.58 ± 0.11	0.54 ± 0.02	59 ± 1.5	202 ± 0.25	138 ± 2.0
F6	100 ± 1.15	3.36 ± 0.13	0.63 ± 0.01	54 ± 2.6	156 ± 1.75	137 ± 4.1
F7	99 ± 0.99	3.20 ± 0.12	0.51 ± 0.06	55 ± 4.3	166 ± 2.17	133 ± 4.3
F8	101 ± 1.56	3.37 ± 0.16	0.67 ± 0.02	55 ± 2.0	166 ± 1.45	134 ± 2.0
F9	100 ± 1.21	3.28 ± 0.08	0.63 ± 0.01	47 ± 2.5	203 ± 1.32	122 ± 2.5

^*^All values represent mean ± standard deviation, *n* = 20; ∗∗all values represent mean ± standard deviation, *n* = 6; ∗∗∗all values represent mean ± standard deviation, *n* = 3.

**Table 4:** Dissolution parameters of MCZ F9 and marketed tablets (Mean ± SD, *n* = 3).

Formulation	(*Q*_30_)	IDR (%/min)	DE	RDR
F9 tablet	98.61 ± 0.25	3.29	63.37	1.51
Marketed tablet	65.43 ± 0.57	2.18	45.53	

**Table 5:** FTIR graph interpretation of pure MCZ and camphor.

Pure MCZ FTIR graph interpretation			Camphor FTIR graph interpretation		
Region in cm^−1^	Type of vibration	Functional group present	Region in cm^−1^	Type of vibration	Functional group present
~2400–2500	NH_3_ stretch	Ammonium ion	2957	CH_3_ stretch	Methyl
1475	C=C stretch	Aromatic (unsaturated)	1448	CH_3_ bending	Methyl
1450	CH_3_ stretch	Methyl	1738	C=O stretch	Ketone
1901	C–N stretch	Nitrile			
698	C–Cl stretch	Carbon-chlorine			

**Table 6:** Stability studies of MCZ F9 fast dissolving tablets (*n* = 3).

Time (min)	Before storage	After 6 months storage	*t*-test at 0.05 LS	Similarity factor (F2)
0	0.00 ± 0.00	0.00 ± 0.00	Not significant	79.16
5	25.67 ± 0.41	23.18 ± 0.93		
10	43.71 ± 0.52	41.63 ± 0.18		
15	81.45 ± 0.32	78.36 ± 0.75		
30	98.61 ± 0.25	96.82 ± 0.34		
**% Assay**	99.94 ± 1.24	98.64 ± 1.18	Not significant	—
